# Supplementary material for: Mycobacterium smegmatis does not display functional redundancy in nitrate reductase enzymes
Source: PLoS One. 2021 Jan 20;16(1):e0245745. doi: 10.1371/journal.pone.0245745 (PMC7816997; doi:10.1371/journal.pone.0245745)
Supplement: S1 Fig — (A) Schematic representation of genomic maps of wild type and mutant narB regions. Restriction enzymes, probes and expected fragment sizes for southern blot confirmation are depicted. (B) Southern blot with upstream probe (US). Lane 1: Marker λIV, Lane 2: Empty, Lane 3: NotI digested wild type DNA, Lane 4: NotI digested ΔnarB DNA, Lane 5: Empty, Lane 6: SacI digested wild type DNA, Lane 7: SacI digested ΔnarB DNA. (C) Southern blot with downstream probe (DS). Lane 1: Marker λIV, Lane 2: Empty, Lane 3: NotI digested wild type DNA, Lane 4: NotI digested ΔnarB DNA, Lane 5: Empty, Lane 6: NcoI digested wild type DNA, Lane 7: NcoI digested ΔnarB DNA. (PDF) [file pone.0245745.s001.pdf]

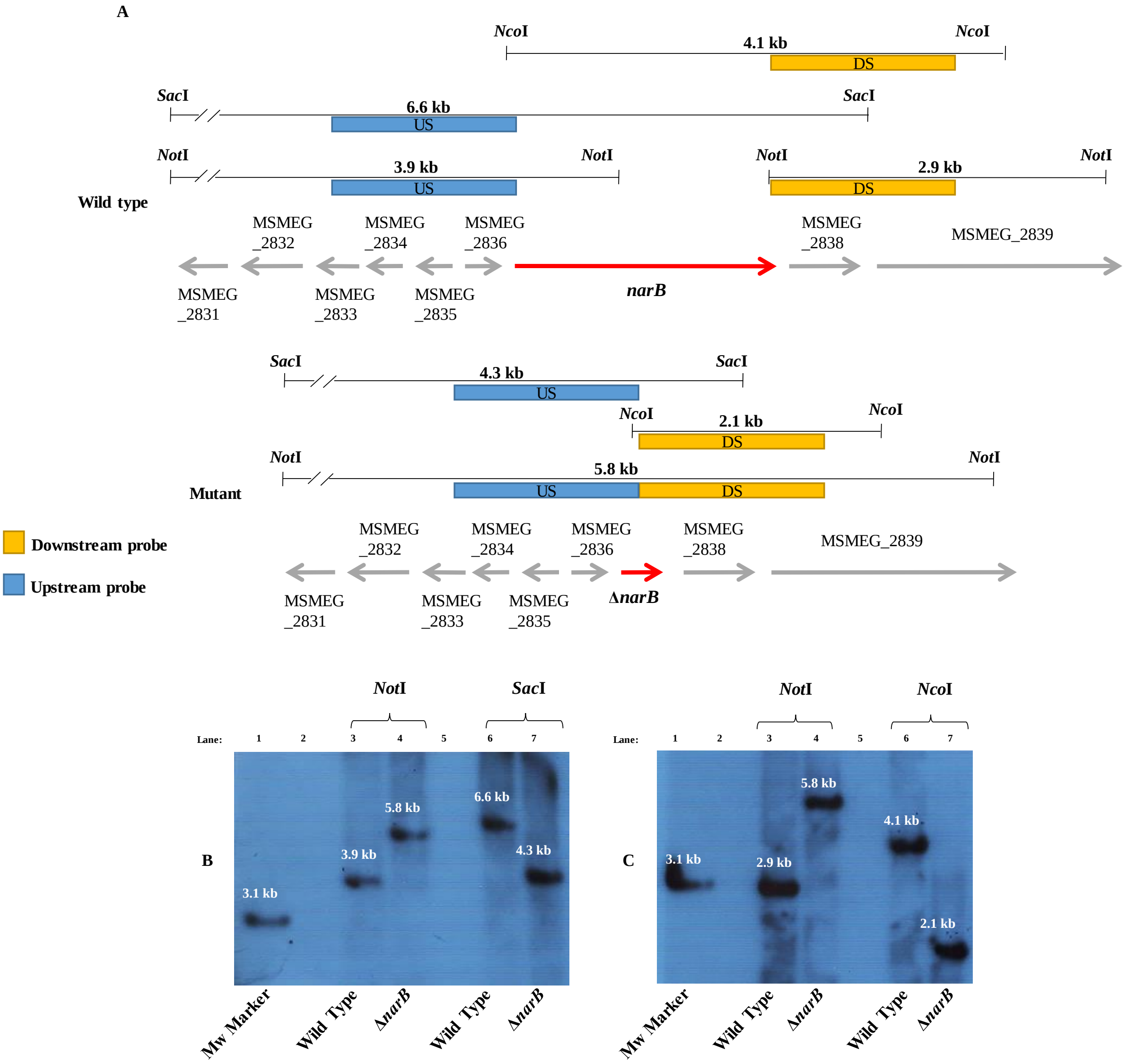

S1 Figure: **Genotypic confirmation of Δ*narB*. (A)** Schematic representation of genomic maps of wild type and mutant *narB* regions. Restriction enzymes, probes and expected fragment sizes for Southern blot confirmation are depicted. Maps are not drawn to scale. **(B)** Southern blot with upstream probe (US). Lane 1: Marker λIV, Lane 2: Empty, Lane 3: *Not*I digested wild type DNA, Lane 4: *Not*I digested Δ*narB* DNA, Lane 5: Empty, Lane 6: *Sac*I digested wild type DNA, Lane 7: *Sac*I digested Δ*narB* DNA. **(C)** Southern blot with downstream probe (DS). Lane 1: Marker λIV, Lane 2: Empty, Lane 3: *Not*I digested wild type DNA, Lane 4: *Not*I digested Δ*narB* DNA, Lane 5: Empty, Lane 6: *Nco*I digested wild type DNA, Lane 7: *Nco*I digested Δ*narB* DNA.
