# Supplementary material for: Mycobacterium smegmatis does not display functional redundancy in nitrate reductase enzymes
Source: PLoS One. 2021 Jan 20;16(1):e0245745. doi: 10.1371/journal.pone.0245745 (PMC7816997; doi:10.1371/journal.pone.0245745)
Supplement: S2 Fig — (A) Schematic representation of genomic maps of wild type and mutant narGHJI regions. Restriction enzymes, probes and expected fragment sizes for southern blot confirmation are depicted. (B) Southern blot with upstream probe (US). Lane 1: Marker λIV, Lane 2: BamHI digested wild type DNA, Lane 3: BamHI digested ΔnarGHJI DNA, Lane 4: BamHI digested ΔnarB ΔnarGHJI DNA. (C) Southern blot with downstream probe (DS). Lane 1: Marker λIV, Lane 2: PstI digested wild type DNA, Lane 3: PstI digested ΔnarGHJI DNA, Lane 4: PstI digested ΔnarB ΔnarGHJI DNA, Lane 5: Empty, Lane 6: MuI digested wild type DNA, Lane 7: MluI digested ΔnarGHJI DNA, Lane 8: MluI digested ΔnarB ΔnarGHJI DNA. (PDF) [file pone.0245745.s002.pdf]

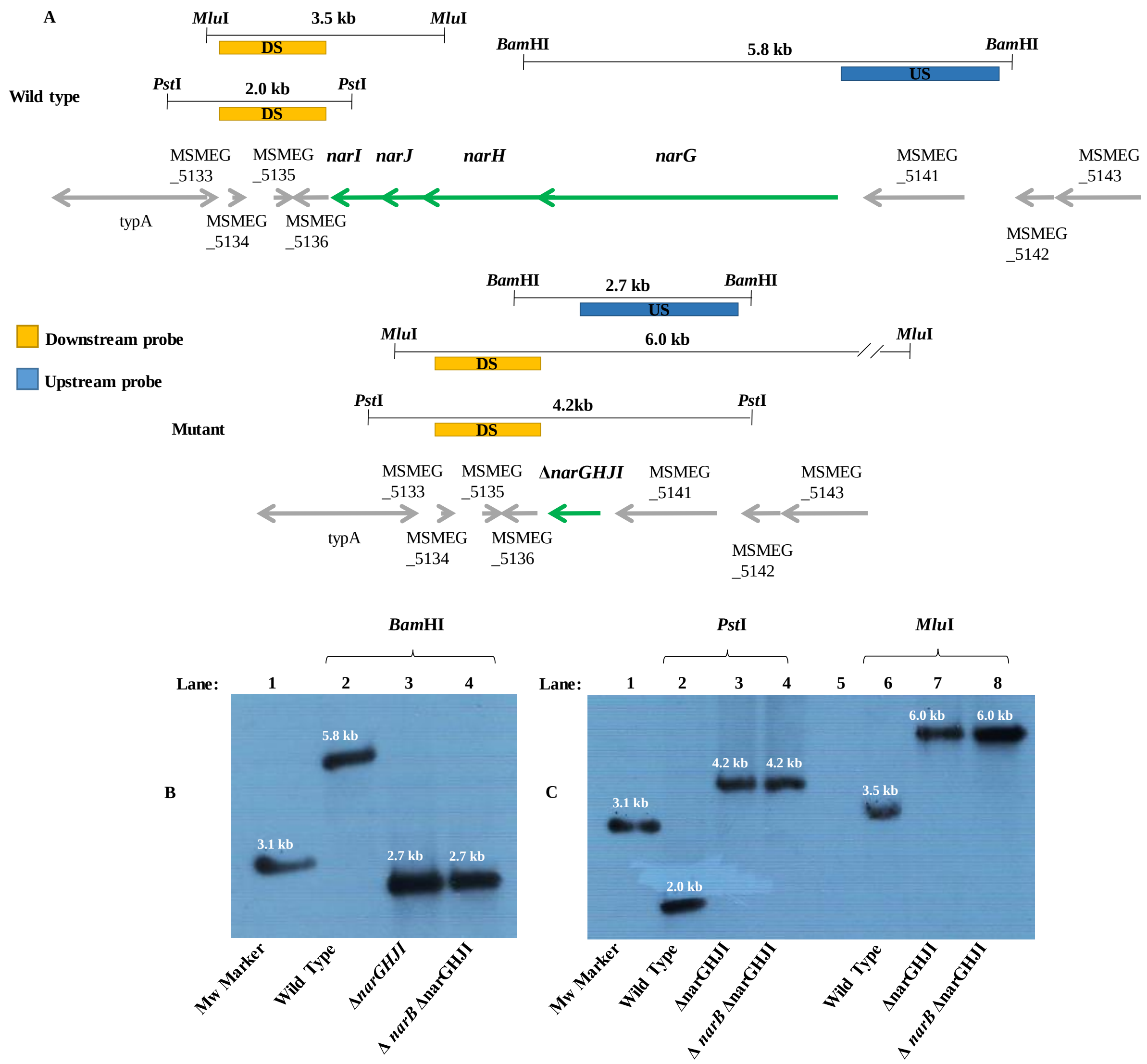

S2 Figure: Genotypic confirmation of Δ*narGHJI* and Δ*narB* Δ*narGHJI*. (A) Schematic representation of genomic maps of wild type and mutant *narGHJI* regions. Restriction enzymes, probes and expected fragment sizes for Southern blot confirmation are depicted. Maps are not drawn to scale. **(B)** Southern blot with upstream probe (US). Lane 1: Marker λIV, Lane 2: *Bam*HI digested wild type DNA, Lane 3: *Bam*HI digested Δ*narGHJI* DNA, Lane 4: *Bam*HI digested Δ*narB* Δ*narGHJI* DNA. **(C)** Southern blot with downstream probe (DS). Lane 1: Marker λIV, Lane 2: *Pst*I digested wild type DNA, Lane 3: *Pst*I digested Δ*narGHJI* DNA, Lane 4: *Pst*I digested Δ*narB* Δ*narGHJI* DNA, Lane 5: Empty, Lane 6: *Mu*I digested wild type DNA, Lane 7: *Mlu*I digested Δ*narGHJI* DNA, Lane 8: *Mlu*I digested Δ*narB* Δ*narGHJI* DNA.
