# Supplementary material for: Mycobacterium smegmatis does not display functional redundancy in nitrate reductase enzymes
Source: PLoS One. 2021 Jan 20;16(1):e0245745. doi: 10.1371/journal.pone.0245745 (PMC7816997; doi:10.1371/journal.pone.0245745)
Supplement: S3 Fig — (A) Schematic representation of genomic maps of wild type and mutant MSMEG_4206 regions. Restriction enzymes, probes and expected fragment sizes for Southern blot confirmation are depicted. Maps are not drawn to scale. (B) Southern blot with upstream probe (US). Lane 1: Marker λIV, Lane 2–4 Acc651 digested DNA from wild type, Δ4206, and the ΔnarB Δ4206 mutant strains respectively; Lanes 5–7: NotI digested DNA from the wild type, Δ4206, and the ΔnarB Δ4206 mutant strains respectively. (PDF) [file pone.0245745.s003.pdf]

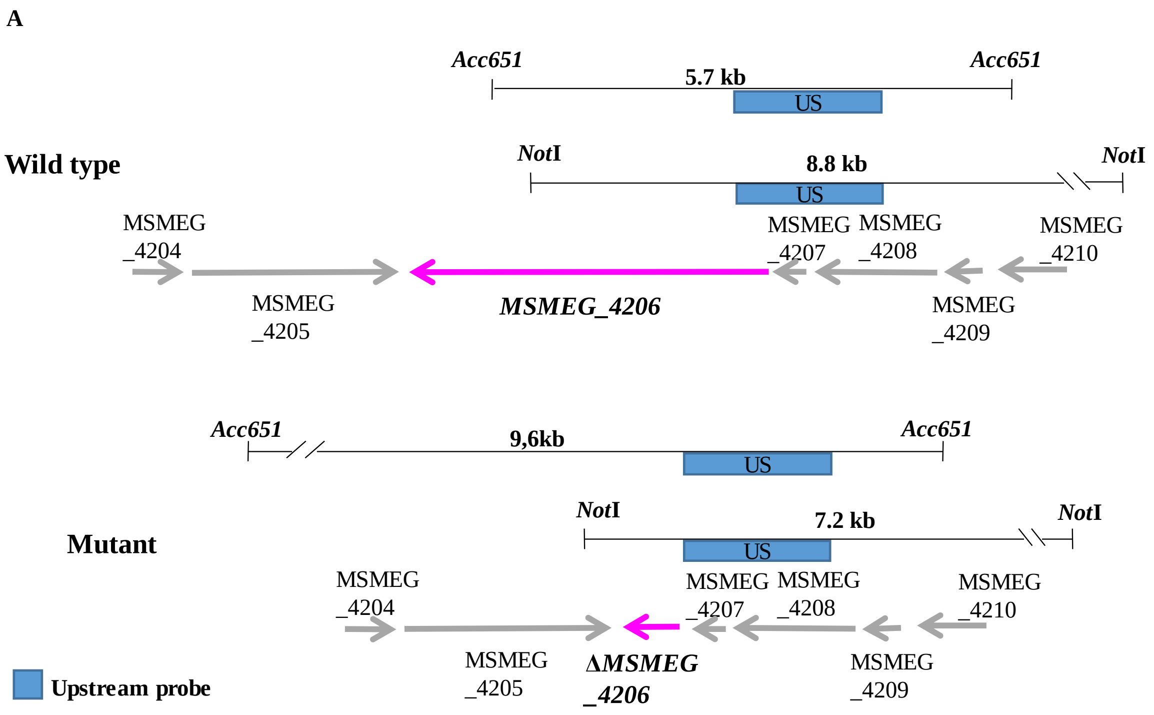

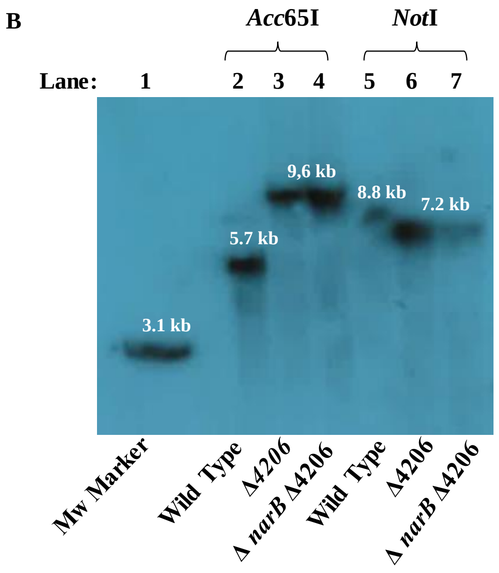

S3 Figure: Southern blot confirmation of MSMEG_4206 mutant strains. **(A)** Schematic representation of genomic maps of wild type and mutant MSMEG_4206 regions. Restriction enzymes, probes and expected fragment sizes for Southern blot confirmation are depicted. Maps are not drawn to scale. **(B)** Southern blot with upstream probe (US). Lane 1: Marker λIV, Lane 2 - 4 *Acc*651 digested DNA from wild type, Δ4206, and the Δ*narB* Δ4206 mutant strains respectively; Lanes 5-7: *Not*I digested DNA from the wild type, Δ4206, and the Δ*narB* Δ4206 mutant strains respectively.
