# Supplementary material for: Mycobacterium smegmatis does not display functional redundancy in nitrate reductase enzymes
Source: PLoS One. 2021 Jan 20;16(1):e0245745. doi: 10.1371/journal.pone.0245745 (PMC7816997; doi:10.1371/journal.pone.0245745)
Supplement: S4 Fig — (A) Schematic representation of genomic maps of wild type and mutant MSMEG_6816 regions. Restriction enzymes, probes and expected fragment sizes for Southern blot confirmation are depicted. Maps are not drawn to scale. (B) Southern blot with upstream probe (US). Lane 1: Marker λIV, Lane 2–4 PstI digested DNA from wild type, Δ6816, and the ΔnarB Δ6816 mutant strains; Lanes 5–7 Acc651 digested DNA from wild type, Δ6816, and the ΔnarB Δ6816 mutant strains. (PDF) [file pone.0245745.s004.pdf]

A

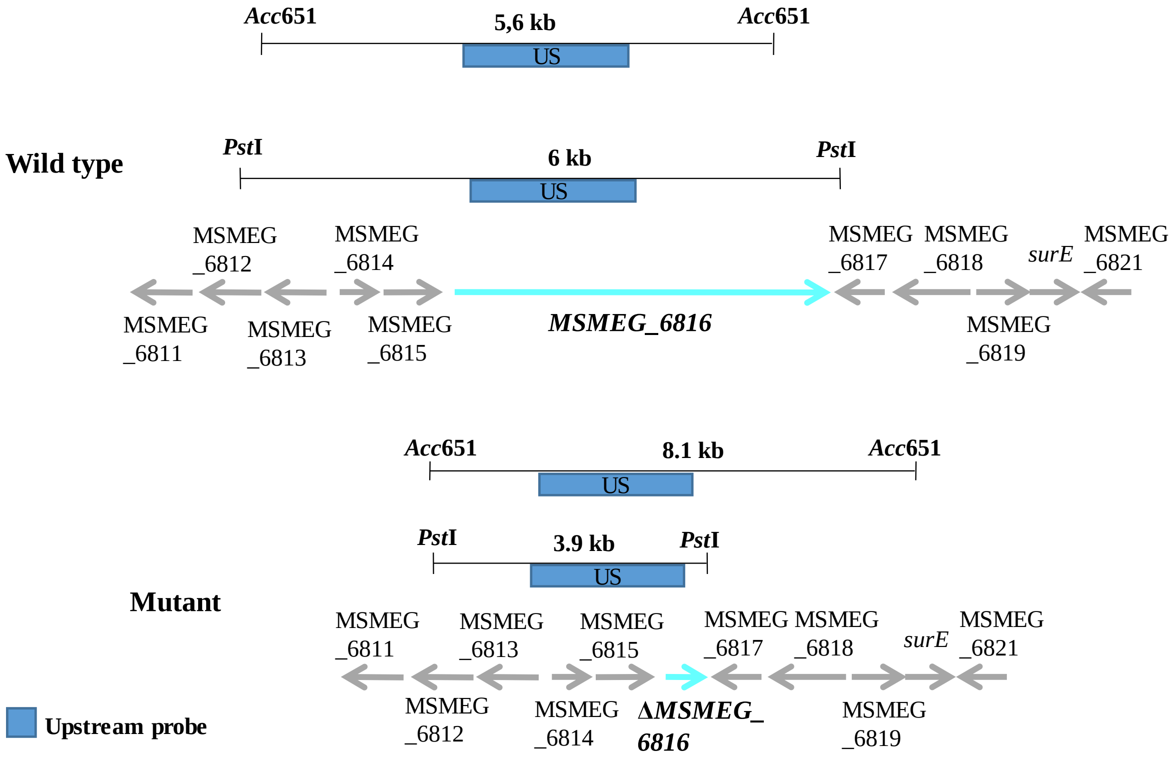

B

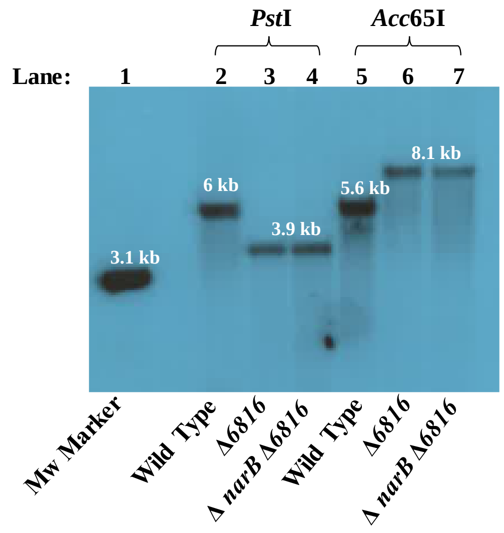

S4 Figure: Southern blot confirmation of MSMEG_6816 mutant strains. **(A)** Schematic representation of genomic maps of wild type and mutant MSMEG_6816 regions. Restriction enzymes, probes and expected fragment sizes for Southern blot confirmation are depicted. Maps are not drawn to scale. **(B)** Southern blot with upstream probe (US). Lane 1: Marker λIV, Lane 2 - 4 *Pst*I digested DNA from wild type, Δ6816, and the Δ*narB* Δ6816 mutant strains; Lanes 5-7 *Acc*651 digested DNA from wild type, Δ6816, and the Δ*narB* Δ6816 mutant strains.
