# Supplementary material for: Mycobacterium smegmatis does not display functional redundancy in nitrate reductase enzymes
Source: PLoS One. 2021 Jan 20;16(1):e0245745. doi: 10.1371/journal.pone.0245745 (PMC7816997; doi:10.1371/journal.pone.0245745)
Supplement: S5 Fig — (A) Schematic representation of genomic maps of wild type and mutant MSMEG_2237 regions. Restriction enzymes, probes and expected fragment sizes for Southern blot confirmation are depicted. Maps are not drawn to scale. (B) Southern blot with upstream probe (US). Lane 1: Marker λIV, Lane 2 and 3 Acc65I digested DNA from wild type and ΔnarB Δ2237 respectively; Lanes 4 and 5 NruI digested DNA from wild type and ΔnarB Δ2237 respectively. (PDF) [file pone.0245745.s005.pdf]

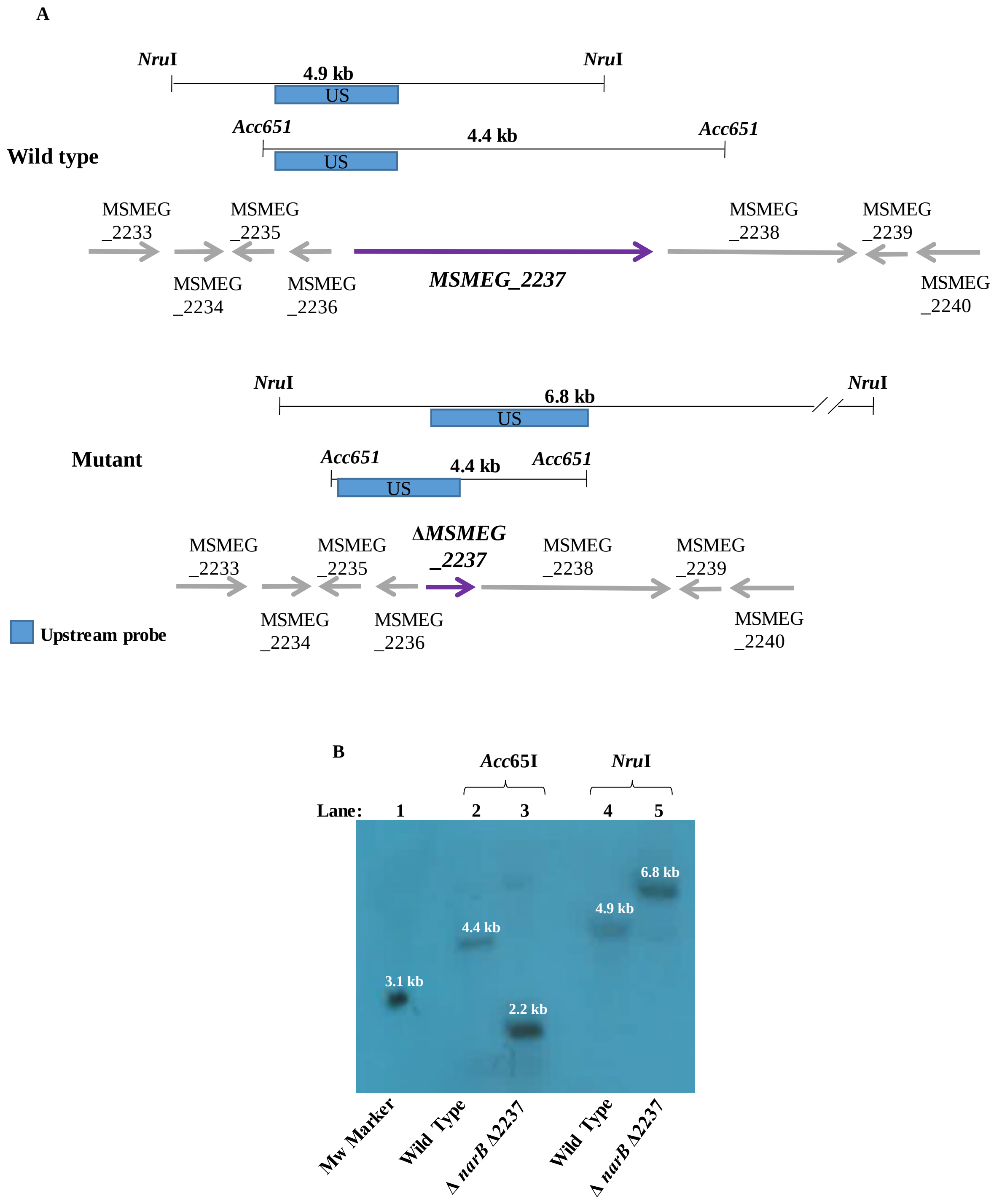

S5 Figure: Southern blot confirmation of MSMEG_2237 mutant strains. **(A)** Schematic representation of genomic maps of wild type and mutant MSMEG_2237 regions. Restriction enzymes, probes and expected fragment sizes for Southern blot confirmation are depicted. Maps are not drawn to scale. **(B)** Southern blot with upstream probe (US). Lane 1: Marker λIV, Lane 2 and 3 *Acc*65I digested DNA from wild type and Δ*narB* Δ2237 respectively; Lanes 4 and 5 *Nru*I digested DNA from wild type and Δ*narB* Δ2237 respectively.
