# Supplementary material for: Mycobacterium smegmatis does not display functional redundancy in nitrate reductase enzymes
Source: PLoS One. 2021 Jan 20;16(1):e0245745. doi: 10.1371/journal.pone.0245745 (PMC7816997; doi:10.1371/journal.pone.0245745)
Supplement: S6 Fig — The average of three independent experiments was plotted for each curve and standard errors are depicted. **** Statistical significance was determined by 2way-ANOVA. (PDF) [file pone.0245745.s006.pdf]

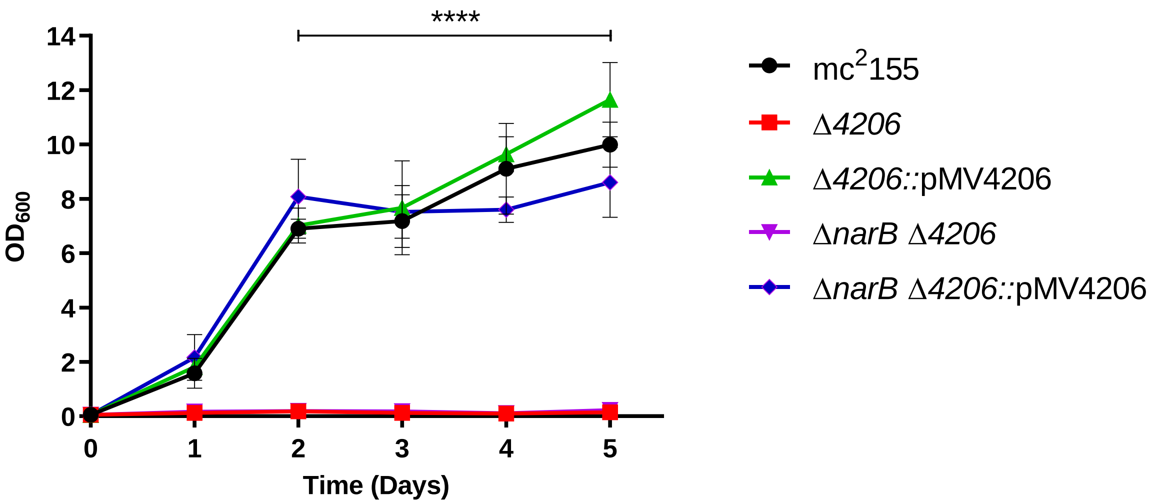

S6 Figure: Complementation of Δ4206 and Δ*narB* Δ4206 with a single copy of *MSMEG_4206* restores growth in MPLN. The average of three independent experiments was plotted for each curve and standard errors are depicted. **** Statistical significance was determined by 2way-ANOVA.
