# Supplementary material for: Mycobacterium smegmatis does not display functional redundancy in nitrate reductase enzymes
Source: PLoS One. 2021 Jan 20;16(1):e0245745. doi: 10.1371/journal.pone.0245745 (PMC7816997; doi:10.1371/journal.pone.0245745)
Supplement: S7 Fig — Over time, the pink colour fades in wild type samples as a result of nitrate depletion via NR activity. A pink colour is maintained throughout the experiment for ΔmoaD2 ΔmoaE2 samples, due to the lack of NR activity in this strain. (PDF) [file pone.0245745.s007.pdf]

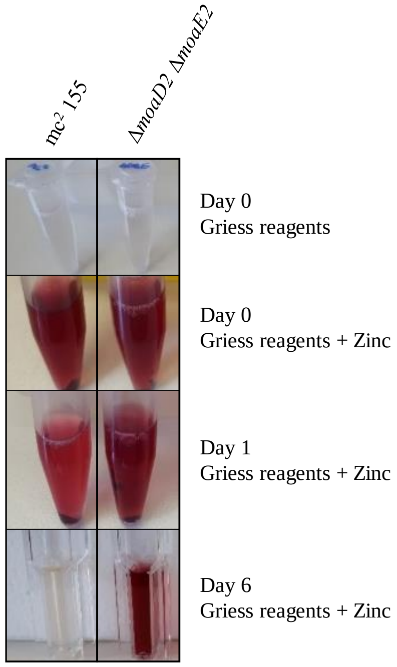

S7 Figure: An example of the visual results obtained for the modified Griess assay. Over time, a less intense pink colour is observed in wild type samples as a result of nitrate depletion via NR activity. A pink colour is maintained throughout the experiment for Δ*moaD2* Δ*moaE2* samples, due to the lack of NR activity in this strain.
