# Supplementary material for: Mycobacterium smegmatis does not display functional redundancy in nitrate reductase enzymes
Source: PLoS One. 2021 Jan 20;16(1):e0245745. doi: 10.1371/journal.pone.0245745 (PMC7816997; doi:10.1371/journal.pone.0245745)
Supplement: S1 Table — (PDF) [file pone.0245745.s008.pdf]

S1 Table: Primers used for gene expression analysis by qRT PCR

Primer name	Sequence 5'→3'	Amplicon (bp)
narG RTF	CAACGGGGTCCTGGTATGTC	159
narG RTR	TTGATCAACAGCCGGGTCAG	
narB RTF3	TTCCACACCCGCACCAAG	200
narB RTR3	AACGGCAGGAACACCACC	
SSF	GGGCGTGATGTCCATCTCCT	121
SSR	GTATCCCGGTGCATGGTC	
2237RTF	GGAAGTGGTGGTTGGCGGAT	150
2237RTR	GCAGTGGAGGTGACAGGTTT	
6816RTF	GAGTCGCCACAGTCCAATCC	151
6816RTR	CGGTGGGATGGATGAAGACC	
4206RTF	CCGATCCGCTATGTCAACGA	244
4206RTR	TTGAGCACCATCGGATAGGC	
