# Supplementary material for: Mycobacterium smegmatis does not display functional redundancy in nitrate reductase enzymes
Source: PLoS One. 2021 Jan 20;16(1):e0245745. doi: 10.1371/journal.pone.0245745 (PMC7816997; doi:10.1371/journal.pone.0245745)
Supplement: S2 Table — (PDF) [file pone.0245745.s009.pdf]

S2 Table: Primers used for the construction of suicide vectors

	Primer name	Sequence 5'-3'	Amplicon (bp)
narB downstream region	narBDSF	GGCGCG**CTG CAG**GCCTGATCCCACTGCTTCT (*Pst*I)	1695
	narBDSR	GGCGCG**AGATCT**CTCTGAGAGGGCCGATCAT (*Bgl*II)	
narB upstream region	narBUSF	GGCGCGC**AGATCT**GGTCTGTGCGAGCCATGAT (*Bgl*II)	1908
	narBUSR	GGCGCG**AAGCTT**GGGGTACAAGCTTGAGGACA (*Hind*III)	
narGHJI upstream region	narUPF	GCCG**AAGCTT**GGACTCTACGACGTGCTCAG (*Hind*III)	1672
	narUPR	GCCG**AGATCT**CAGCAGTTCTTCCACACGTC (*Bgl*II)	
narGHJI downsream region	narDF	GCCG**AGATCT**CGGCTGGTGACAAGAAGG (*Bgl*II)	1119
	narDR	GCCG**CTGCAG**GTGATTCTCGCAGGTAGTCGAG (*Pst*I)	
MSMEG_4206 upstream region	4206USF	GCGC**CATATG**GTTGGCCATGTGGCCATG (*Nde*I)	1195
	4206USR	GCGC**GGTACC**AAGACGAGTTCGGCAACG (*Acc*651)	
MSMEG_4206 downstream region	4206DSF	GCGC**AAGCTT**CGCCGAACGTGTGCTCGA (*Hind*III)	1203
	4206DSR	GCGC**CATATG**TACTGAGCCGATTACCGGGGA (*Nde*I)	
MSMEG_6816 upstream region	6816USF	GCGC**AAGCTT**TCGTGCGGTTGACCGATA (*Hind*III)	1112
	6816USR	GCGC**GAATTC**CTTGAGTGTTTGCATGGG (*Eco*RI)	
MSMEG_6816 downstream region	6816DSF	GCGC**GAATTC**TCGGCGTGAGCATCAACG (*Eco*RI)	1110
	6816DSR	GCGC**GGTACC**CGGCCGTCATGCAGTTCA (*Acc*651)	
MSMEG_2237 upstream region	2237USF	GCGC**AAGCTT**CGAGCAGCTTCTCCTGGT (*Hind*III)	1101
	2237USR	GCGC**CCATGG**TCCGCTCTCGATCACCAC (*Nco*I)	
MSMEG_2237 downstream region	2237DSF	GCGC**CCATGG**GGGAGTTCTGGGTGAGTG (*Nco*I)	1098
	2237DSR	GCGC**ACGCGT**GCTCACACGCATCGAGCA (*Mlu*I)	

Restriction sites indicated are those used for cloning
