# Supplementary material for: Mycobacterium smegmatis does not display functional redundancy in nitrate reductase enzymes
Source: PLoS One. 2021 Jan 20;16(1):e0245745. doi: 10.1371/journal.pone.0245745 (PMC7816997; doi:10.1371/journal.pone.0245745)
Supplement: S3 Table — (PDF) [file pone.0245745.s010.pdf]

Table S3: Plasmids and strains used and generated during this study

**Name**	**Description**	**Source/ reference**
<u>Plasmids</u>		
p2NIL	Cloning vector; Kan^r	(1)
pGOAL19	Plasmid carrying *hyg, lacZ, and sacB* genes as a *Pac*I cassette; Amp^r, Hyg^r	(1)
pΔnarB	Knock-out vector for creating unmarked deletion in *M. smegmatis narB*, constructed by cloning PCR-amplified upstream and downstream regions of *narB* in p2NIL and insertion of the *hyg*-*lacZ*-*sacB* cassette from pGOAL19; Kan^r Hyg^r	This work
pΔnarGHJI	Knock-out vector for creating unmarked deletion in *M. smegmatis narGHJI* operon, constructed by cloning PCR-amplified upstream and downstream regions of *narGHJI* operon in p2NIL and insertion of the *hyg*-*lacZ*-*sacB* cassette from pGOAL19; Kan^r Hyg^r	This work
pΔ2237	Knock-out vector for creating unmarked deletion in MSMEG_2237, constructed by cloning PCR-amplified upstream and downstream regions of MSMEG_2237 in p2NIL and insertion of the *lacZ*-*sacB* cassette from pGOAL19; Kan^r	This work
pΔ6816	Knock-out vector for creating unmarked deletion in MSMEG_6816, constructed by cloning PCR-amplified upstream and downstream regions of MSMEG_6816 in p2NIL and insertion of the *lacZ*-*sacB* cassette from pGOAL17; Kan^r	This work
pΔ4206	Knock-out vector for creating unmarked deletion in MSMEG_4206, constructed by cloning PCR-amplified upstream and downstream regions of MSMEG_4206 in p2NIL and insertion of the *hyg*-*lacZ*-*sacB* cassette from pGOAL19; Kan^r Hyg^r	This work
<u>*M. smegmatis* strains</u>		
mc^{2}155	*ept-1* (efficient plasmid transformation) mutant of mc^{2}6	(2)
Δ*narB*	Derivative of mc^{2}155 carrying an unmarked deletion in *M. smegmatis narB*	This work
Δ*narGHJI*	Derivative of mc^{2}155 carrying an unmarked deletion in *M. smegmatis narGHJI* operon	This work
Δ*narGHJI* Δ*narB*	Derivative of Δ*narGHJI* carrying an unmarked deletion in *M. smegmatis* Δ*narB*	This work
Δ*moaD2* Δ*moaE2*	Derivative of *M. smegmatis ΔmoaE2* carrying an unmarked deletion in the *M. smegmatis* $moaD_2$ gene	(3)
Δ4206	Derivative of mc^{2}155 carrying an unmarked deletion in *M. smegmatis* MSMEG_4206	This work
Δ*narB* Δ*4206*	Derivative of Δ*narB* carrying an unmarked deletion in *M. smegmatis* MSMEG_4206	This work
Δ6816	Derivative of mc^{2}155 carrying an unmarked deletion in *M. smegmatis* MSMEG_6816	This work
Δ*narB* Δ*6816*	Derivative of Δ*narB* carrying an unmarked deletion in *M. smegmatis* MSMEG_6816	This work
Δ*narB* Δ*2237*	Derivative of Δ*narB* carrying an unmarked deletion in *M. smegmatis* MSMEG_2237	This work

References

1. Parish T, Stoker NG. Use of a flexible cassette method to generate a double unmarked *Mycobacterium tuberculosis tlyA plcABC* mutant by gene replacement. Microbiology. 2000;146 (Pt 8):1969-75.

2. Snapper SB, Melton RE, Mustafa S, Kieser T, Jacobs WR, Jr. Isolation and characterization of efficient plasmid transformation mutants of *Mycobacterium smegmatis*. Mol Microbiol. 1990;4(11):1911-9.

3. Williams MJ, Kana BD, Mizrahi V. Functional analysis of molybdopterin biosynthesis in mycobacteria identifies a fused molybdopterin synthase in *Mycobacterium tuberculosis*. J Bacteriol. 2011;193(1):98-106.
