# Supplementary material for: Mycobacterium smegmatis does not display functional redundancy in nitrate reductase enzymes
Source: PLoS One. 2021 Jan 20;16(1):e0245745. doi: 10.1371/journal.pone.0245745 (PMC7816997; doi:10.1371/journal.pone.0245745)
Supplement: S4 Table — (PDF) [file pone.0245745.s011.pdf]

S4 Table: Primers for MSMEG_4206 complementation

Primer name	Sequence 5’ → 3’
4206F	GCGC**TGTACA**AACCTGTGCTCGCGGTCAAG (*Bsr*GI)
4206R	GCGC**AAGCTT**TCAGTACACGTCGCGCACGTA (*Hind*III)
