# Supplementary material for: Mycobacterium smegmatis does not display functional redundancy in nitrate reductase enzymes
Source: PLoS One. 2021 Jan 20;16(1):e0245745. doi: 10.1371/journal.pone.0245745 (PMC7816997; doi:10.1371/journal.pone.0245745)
Supplement: S5 Table — (PDF) [file pone.0245745.s012.pdf]

S5 Table: Primers used to sequence MSMEG_4206 frameshift

Primer name	Sequence 5’ → 3’
F4206	CATCTGCACACCGAAGCC
Seq4206R	GACGGGTTGAGCTTGTTGAG
