# Supplementary material for: Mycobacterium smegmatis does not display functional redundancy in nitrate reductase enzymes
Source: PLoS One. 2021 Jan 20;16(1):e0245745. doi: 10.1371/journal.pone.0245745 (PMC7816997; doi:10.1371/journal.pone.0245745)
Supplement: S1 Raw images — (PDF) [file pone.0245745.s013.pdf]

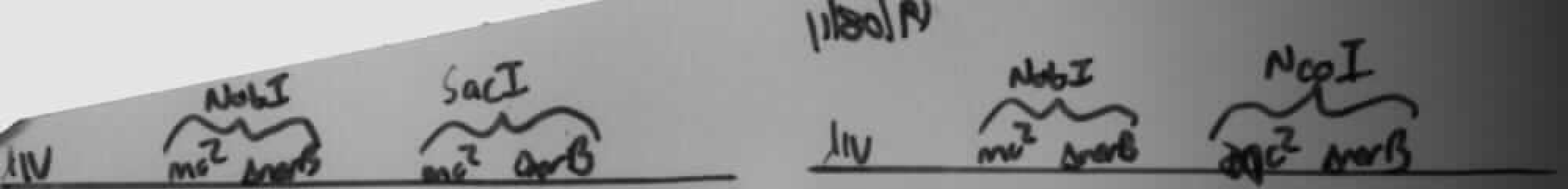

Genotypic confirmation of ΔnarB. (A) Southern blot with upstream probe. Lane 1: Marker λIV, Lane 2: Empty, Lane 3: NotI digested wild type DNA, Lane 4: NotI digested ΔnarB DNA, Lane 5: Empty, Lane 6: SacI digested wild type DNA, Lane 7: SacI digested ΔnarB DNA. (B) Southern blot with downstream probe. Lane 1: Marker λIV, Lane 2: Empty, Lane 3: NotI digested wild type DNA, Lane 4: NotI digested ΔnarB DNA, Lane 5: Empty, Lane 6: NcoI digested wild type DNA, Lane 7: NcoI digested ΔnarB DNA. Blot was transferred from an 8-well, 0.8% agarose gel. Exposure: Kodak Xray film was placed onto membrane previously incubated with CSPD (Roche) and exposed for ~ 1 hour, Films were developed with Axim Fixer and Developer solutions. The blot image was captured using GeneSnap on a Gbox with a white light filter. Blot A and B were cropped and are included in S1 Fig, panel B and C respectively.

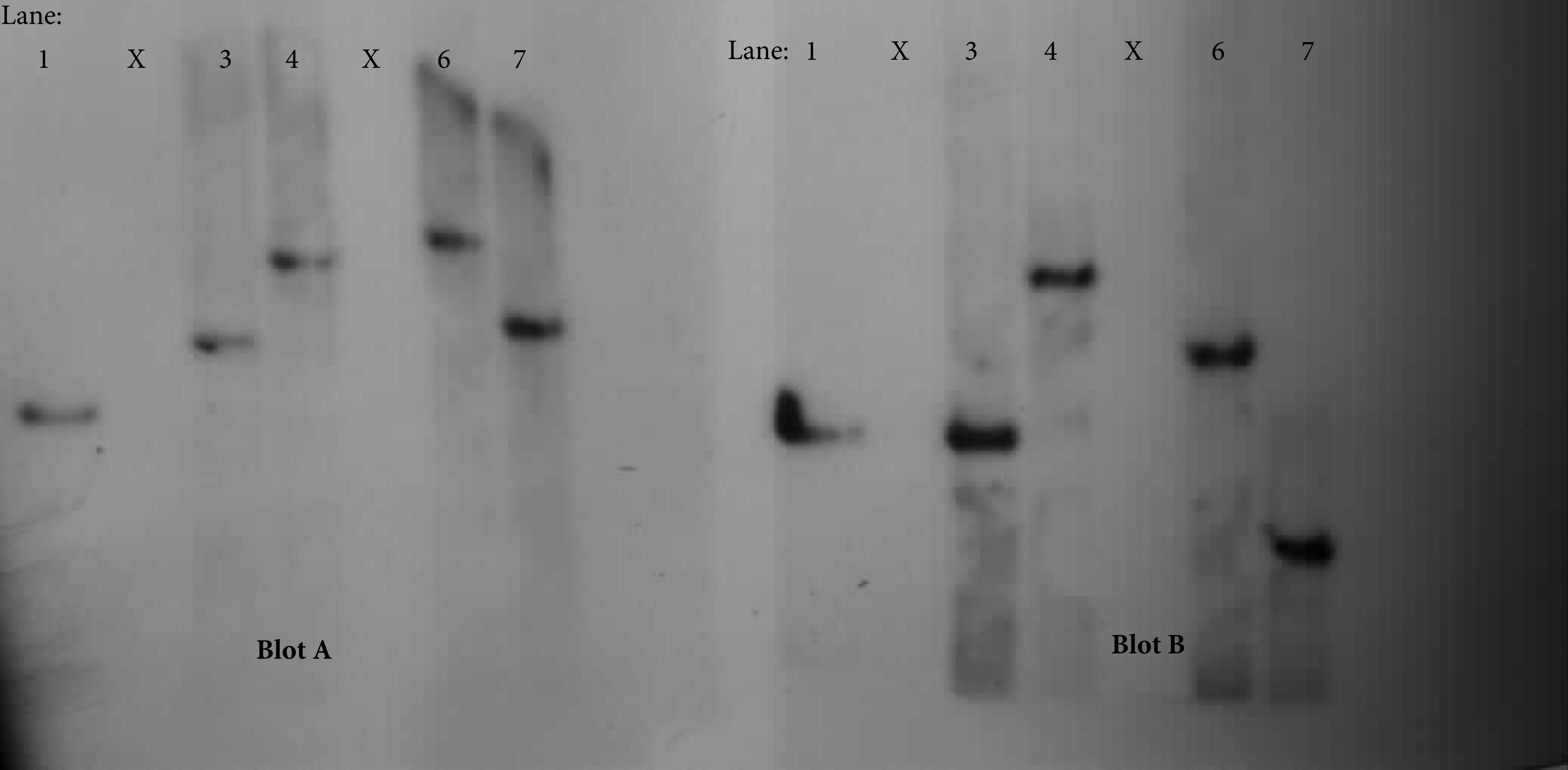

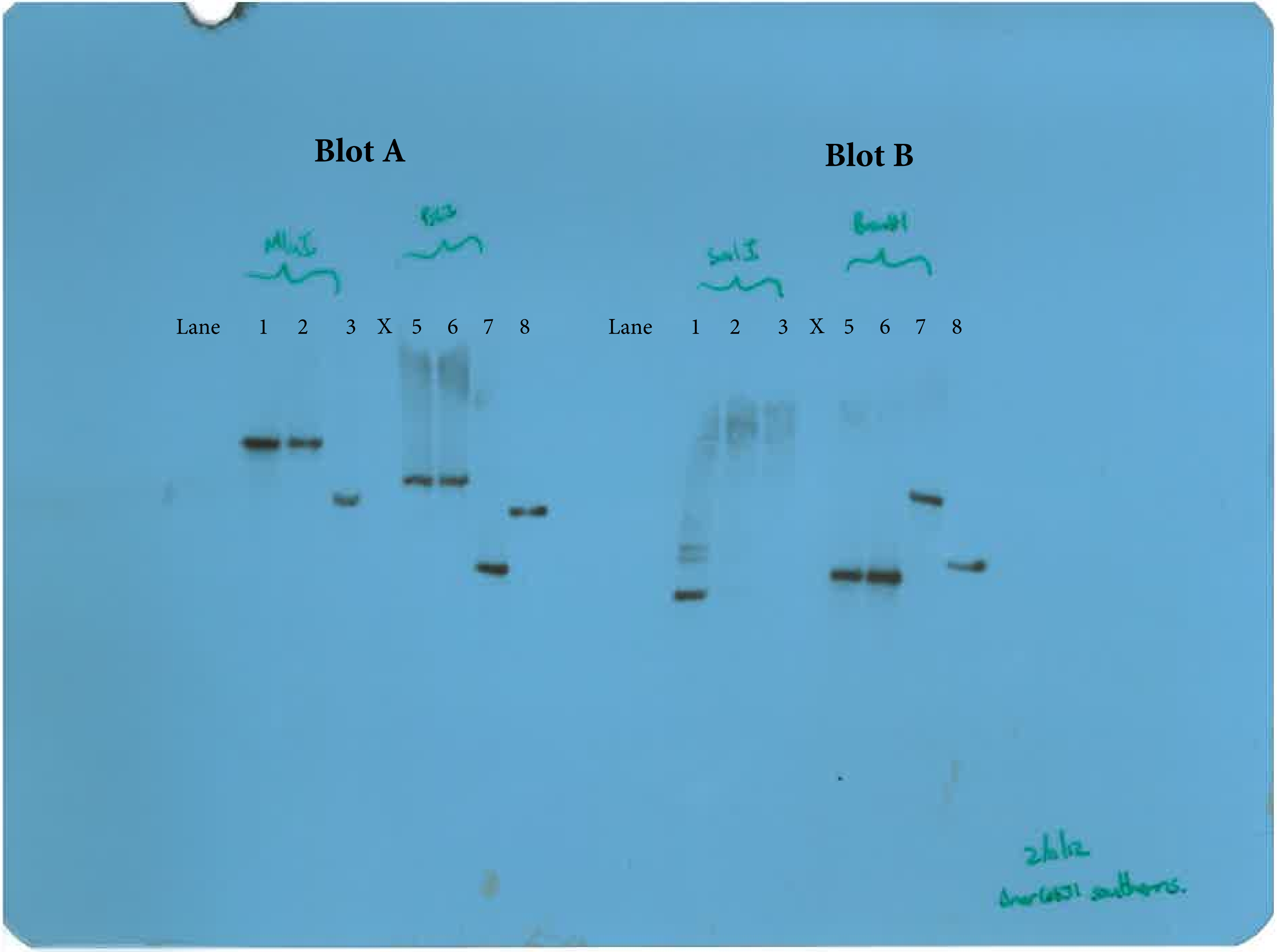

Genotypic confirmation of ΔnarGHJI and ΔnarB ΔnarGHJI. (Blot A) Southern blot with downstream probe . Lane 1: MluI digested ΔnarB ΔnarGHJI DNA, Lane 2: MluI digested ΔnarGHJI DNA, Lane 3: MuI digested wild type DNA, Lane 4: Empty, Lane 5: PstI digested ΔnarB ΔnarGHJI DNA, Lane 6: PstI digested ΔnarGHJI DNA, Lane 7: PstI digested wild type DNA, Lane 8: Marker λIV. (Blot B) Southern blot with upstream probe. Lane 1: SalI digested ΔnarB ΔnarGHJI DNA, Lane 2: SalI digested ΔnarGHJI DNA,PstI digested wild type DNA, Lane 3: SalI digested wild type DNA, Lane 4: Empty, Lane 5: BamHI digested ΔnarB ΔnarGHJI DNA, Lane 6: BamHI digested ΔnarGHJI DNA, Lane 7: BamHI digested wild type DNA, Lane 8: Marker λIV. Blot was transferred from an 8-well, 0.8% agarose gel. Exposure: Kodak Xray film was placed onto membrane previously incubated with CSPD (Roche) and exposed for ~ 1 hour, Films were developed with Axim Fixer and Developer solutions. Developed Xray films were scanned on a Konica Minolta Bizhub C220. Blot A was cropped and included in S2 Fig, panel B. Blot B was cropped to depict Lanes 5-8 and was included in S2 Fig, panel A.

Southern blot confirmation of MSMEG_4206 mutant strains
Southern blot with upstream probe (US). Lane 1: Marker λIV, Lane 2: empty, Lane 3- 5 Acc651 digested DNA from wild type, Δ4206, and the ΔnarB Δ4206 mutant strains respectively; Lanes 6-8: NotI digested DNA from the wild type, Δ4206, and the ΔnarB Δ4206 mutant strains respectively. Exposure: XRay film was placed on top of membrane in a sealed cassette for ~60 minutes. Film was then developed manually in developer and fixer solutions. Blot was cropped and represented in S3 Fig, panel B.

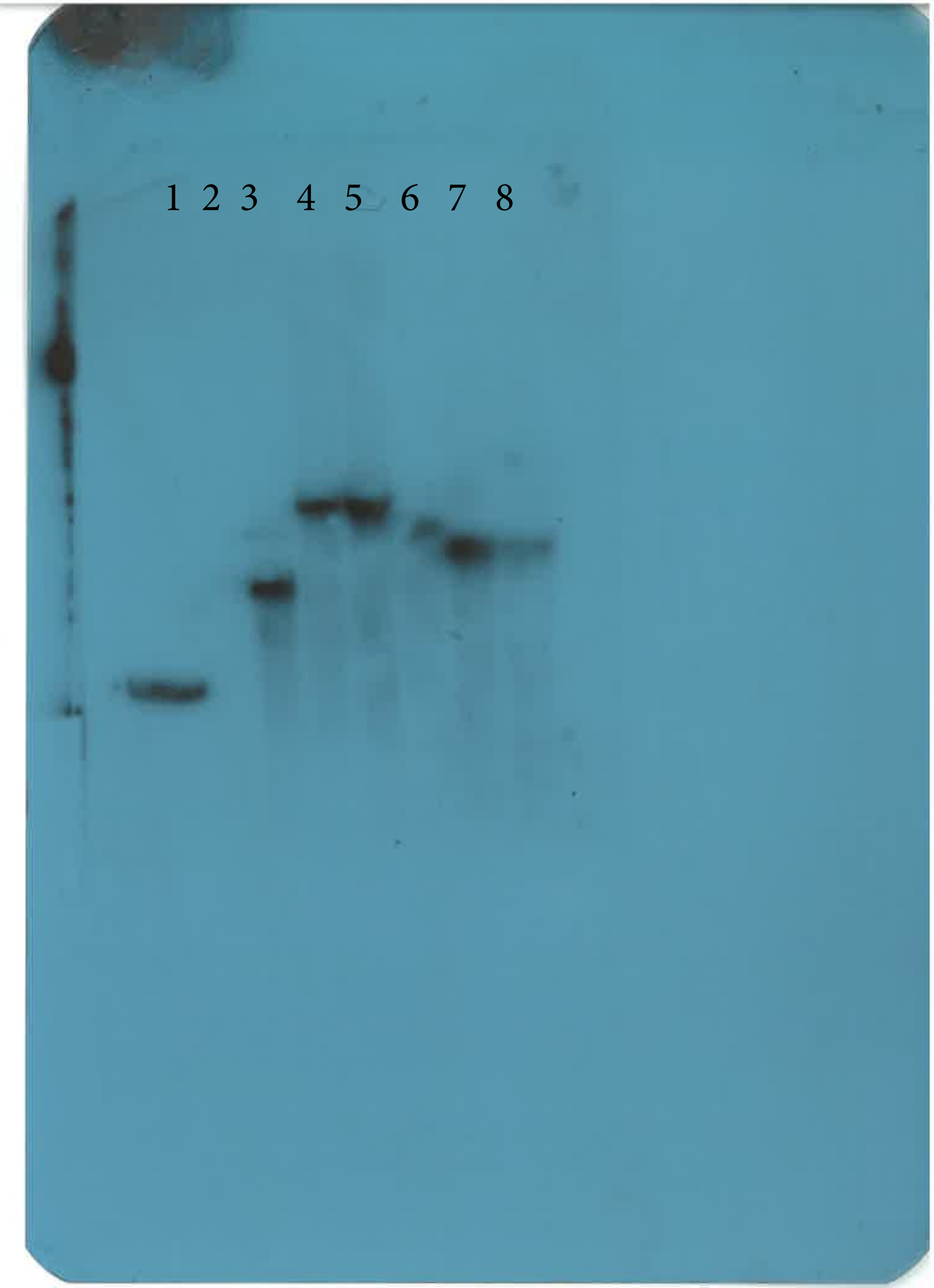

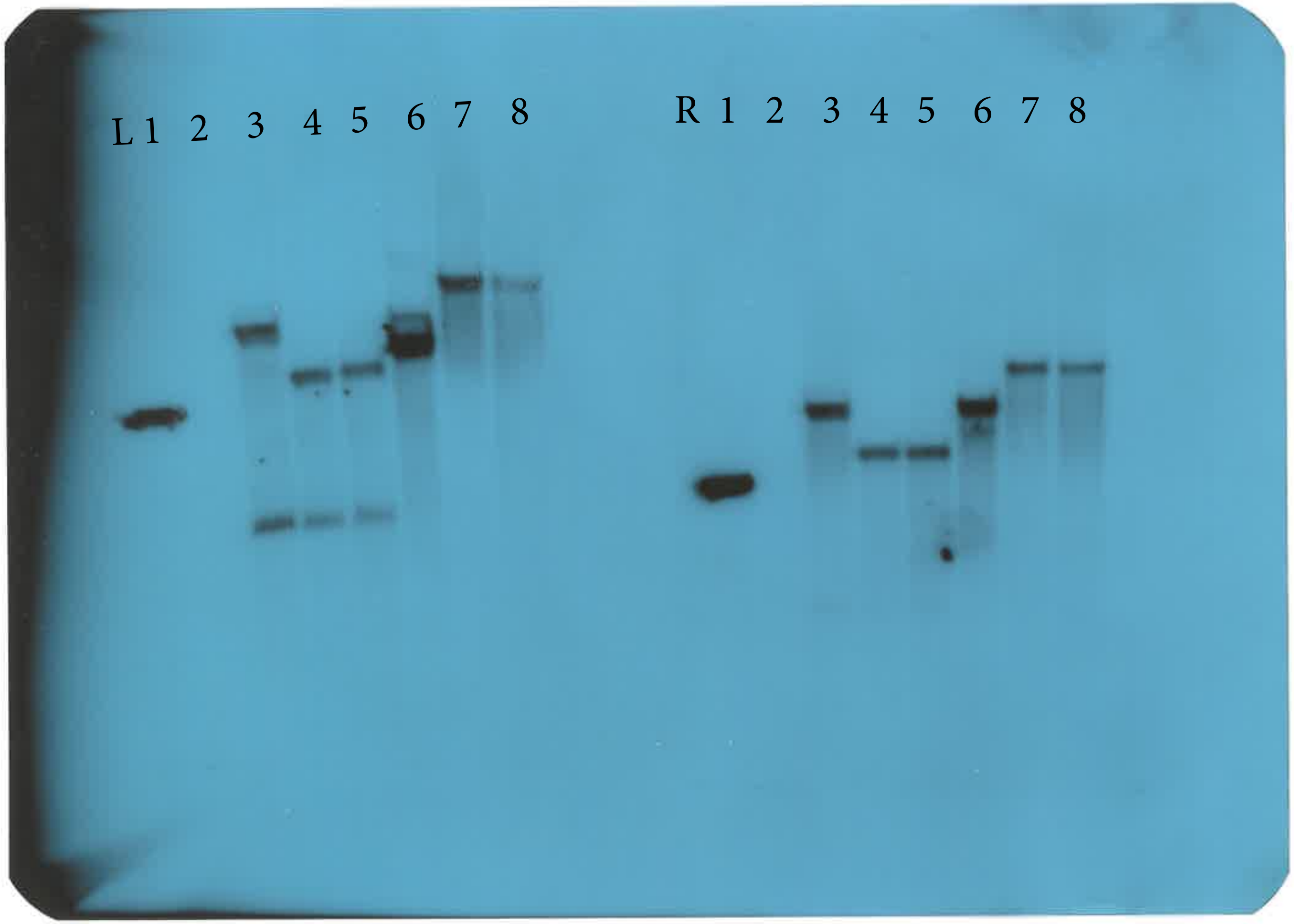

Southern blot confirmation of MSMEG_6816 mutant strains. Left (L): Not applicable; Right (R): Southern blot with upstream probe (US). Lane 1: Marker λIV, Lane 2 empty, Lane 3-5 PstI digested DNA from wild type, Δ6816, and the ΔnarB Δ6816 mutant strains; Lanes 6-8 Acc651 digested DNA from wild type, Δ6816, and the ΔnarB Δ6816 mutant strains. Blot transferred from 8-well, 0.8% agarose gel. Exposure: XRay film was placed on top of membrane in a sealed cassette for ~60 minutes. Film was developed manually in developer and fixer solutions. Right blot was cropped and represented in S4 Fig, panel B.

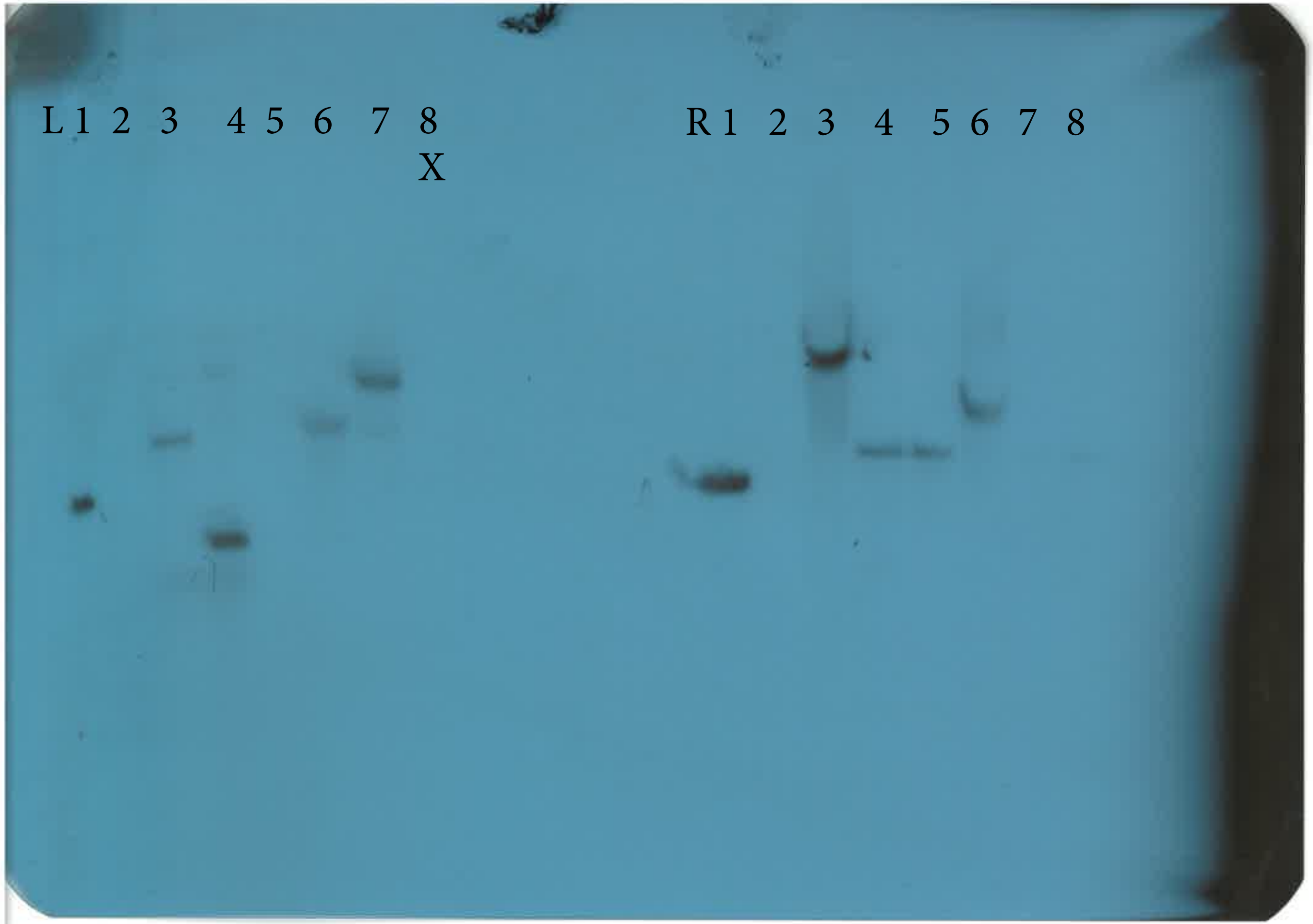

Southern blot confirmation of MSMEG_2237 mutant strains. Left (L): Southern blot with upstream probe (US). Lane 1: Marker λIV, Lane 2: empty, Lane 3 and 4: Acc65I digested DNA from wild type and ΔnarB Δ2237 respectively; Lane 5: empty; Lanes 6 and 7 NruI digested DNA from wild type and ΔnarB Δ2237 respectively; Lane 8: empty. Right (R): not applicable. Blot transferred from 8-well, 0.8% agarose gel. Exposure: XRay film was placed on top of membrane in a sealed cassette for ~60 minutes. Film was developed manually using developer and fixer solutions. Left blot was cropped and represented in Q3 Fig, panel B.
